# Mosaic expression of *Atrx* in the mouse central nervous system causes memory deficits

**DOI:** 10.1242/dmm.027482

**Published:** 2017-02-01

**Authors:** Renee J. Tamming, Jennifer R. Siu, Yan Jiang, Marco A. M. Prado, Frank Beier, Nathalie G. Bérubé

**Affiliations:** 1 Division of Genetics and Development, Children's Health Research Institute, London, Ontario N6C 2V5, Canada; 2 Departments of Paediatrics, Biochemistry and Oncology, Schulich School of Medicine and Dentistry, the University of Western Ontario, Victoria Research Laboratories, London, Ontario N6A 3K7, Canada; 3 Department of Physiology and Pharmacology, Schulich School of Medicine and Dentistry, the University of Western Ontario, London, Ontario N6A 3K7, Canada; 4 Department of Anatomy and Cell Biology and Robarts Research Institute, the University of Western Ontario, London, Ontario N6A 3K7, Canada

**Keywords:** ATRX, Central nervous system, Mouse models, Neurobehaviour

## Abstract

The rapid modulation of chromatin organization is thought to play a crucial role in cognitive processes such as memory consolidation. This is supported in part by the dysregulation of many chromatin-remodelling proteins in neurodevelopmental and psychiatric disorders. A key example is *ATRX*, an X-linked gene commonly mutated in individuals with syndromic and nonsyndromic intellectual disability. The consequences of *Atrx* inactivation for learning and memory have been difficult to evaluate because of the early lethality of hemizygous-null animals. In this study, we evaluated the outcome of brain-specific *Atrx* deletion in heterozygous female mice. These mice exhibit a mosaic pattern of ATRX protein expression in the central nervous system attributable to the location of the gene on the X chromosome. Although the hemizygous male mice die soon after birth, heterozygous females survive to adulthood. Body growth is stunted in these animals, and they have low circulating concentrations of insulin growth factor 1. In addition, they are impaired in spatial, contextual fear and novel object recognition memory. Our findings demonstrate that mosaic loss of ATRX expression in the central nervous system leads to endocrine defects and decreased body size and has a negative impact on learning and memory.

## Abstract (precis)

**Summary:** Heterozygous expression of the X-linked gene *Atrx* in the mouse brain causes deficits in spatial, contextual fear and object recognition memory.

## INTRODUCTION

Alpha thalassemia mental retardation, X-linked, or ATR-X syndrome, is an intellectual disability (ID) disorder that arises from mutations in the *ATRX* gene (OMIM 301040). This rare syndrome is characterized by severe developmental delay, hypotonia, mild α-thalassemia and moderate-to-severe ID (Gibbons et al., 1995). A recent study screened a cohort of nearly 1000 individuals with ID using targeted next-generation sequencing and identified *ATRX* variants as one of the most common causes of ID, reinforcing its importance in cognition (Grozeva et al., 2015). The ATRX protein is a SWI/SNF-type chromatin remodeller. The N-terminal region of the protein contains a histone reader domain that mediates interaction of the protein with histone H3 trimethylated at lysine 9 (H3K9me3) and unmethylated at lysine 4 (H3K4me0) (Dhayalan et al., 2011). A SWI/SNF2-type helicase domain is located in the C-terminal half of the protein and confers ATP-dependent chromatin remodelling activity (Aapola et al., 2000; Gibbons et al., 1997; Picketts et al., 1996). Several proteins have been shown to interact with ATRX, including MeCP2, HP1α, EZH2 and DAXX (Bérubé et al., 2000; Cardoso et al., 1998; Nan et al., 2007; Xue et al., 2003). DAXX is a histone chaperone for histone variant H3.3. In association with ATRX, DAXX deposits H3.3-containing nucleosomes at telomeres and pericentromeric heterochromatin (Drane et al., 2010; Lewis et al., 2010).

Several studies have previously implicated ATRX in the regulation of gene expression through a variety of mechanisms. Chromatin immunoprecipitation (ChIP) sequencing for ATRX in human erythroblasts showed that the protein tends to bind GC-rich regions with high tendency to form G-quadruplexes. For example, ATRX was found to bind tandem repeats within the human α-globin gene cluster, and it was suggested that reduced expression of α-globin might be caused by replication-dependent mechanisms that would affect the expression of nearby genes (Law et al., 2010). The induction of replication stress was in fact detected *in vivo* upon inactivation of *Atrx* in either muscle or brain (Leung et al., 2013; Watson et al., 2013). More recently, our group demonstrated that loss of ATRX corresponds to decreased H3.3 incorporation and increased PolII occupancy in GC-rich gene bodies, including *Neuroligin 4*, an autism susceptibility gene (Levy et al., 2015).

Although the mechanisms by which ATRX modulates chromatin and genes is starting to be resolved, its function in neurons and cognitive processes is still obscure. To address this question, we generated mice with conditional inactivation of *Atrx* in the central nervous system (CNS) starting at early stages of neurogenesis. Although hemizygous male progeny died shortly after birth, heterozygous female mice (henceforth called *Atrx*-cHet), which exhibit mosaic expression of ATRX caused by random X-inactivation, survived to adulthood, allowing the investigation of neurobehavioural outcomes upon inactivation of *Atrx* in the brain.

## RESULTS

### Survival to adulthood depends on the extent of *Atrx* deletion in the CNS

**Fig. 1.**
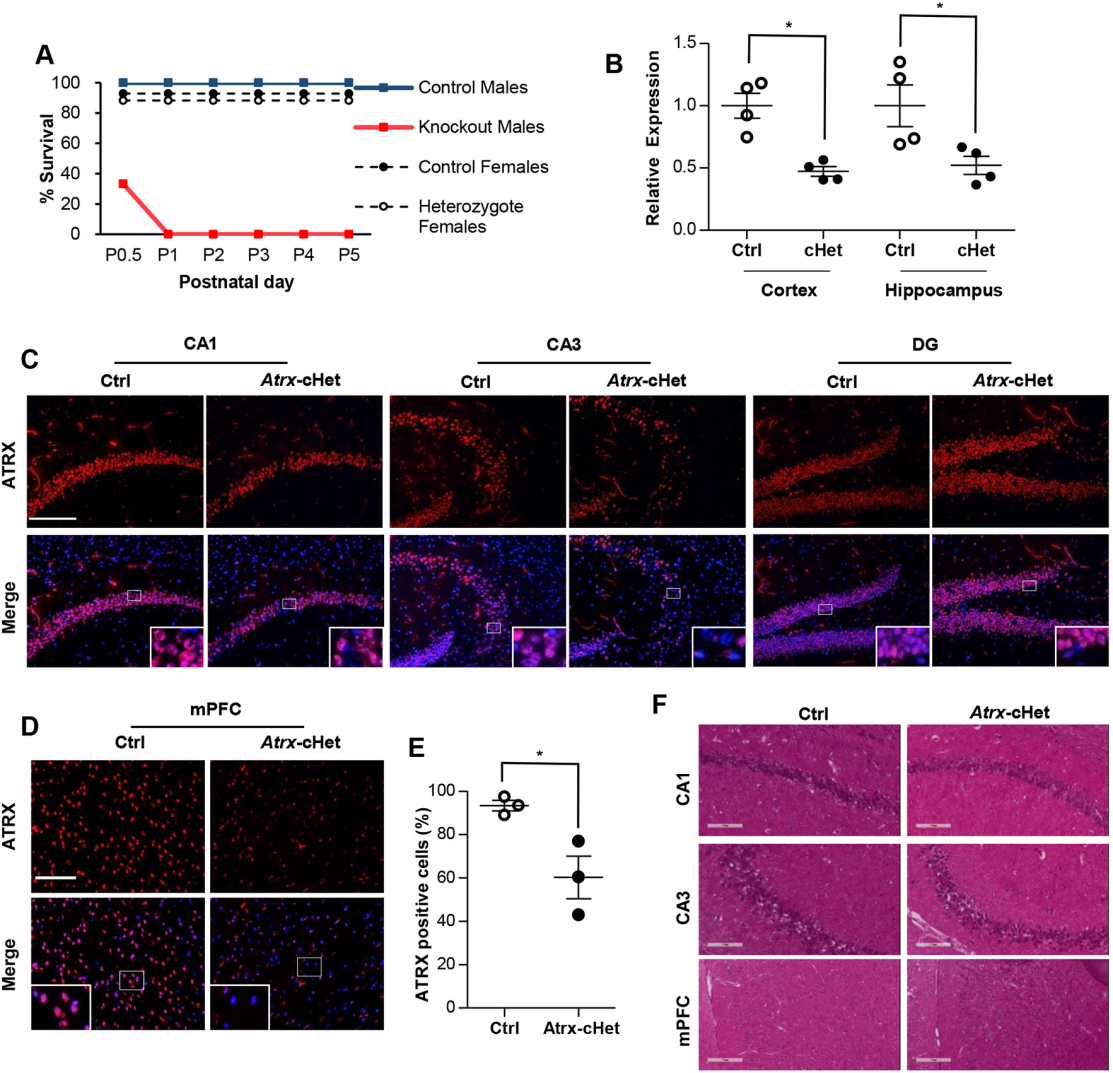
**Mosaic pattern of ATRX expression in the brain of *Atrx*-cHet mice.** (A) Graph depicting the survival of control male mice (*n*=20), knockout male mice (*n*=6), control female mice (*n*=14) and heterozygote female mice (*n*=17) as the percentage survival at each time point. (B) RT-qPCR of *Atrx* (normalized to *Gapdh* expression) in the hippocampus and cortex of *Atrx*-cHet mice and littermate-matched controls (mean±s.e.m. of *n*=4 pairs, Student's two-tailed unpaired *t*-test). *P<0.05. (C) Immunofluorescence staining of ATRX (red) and DAPI (blue) in the hippocampus of control and *Atrx*-cHet mice. Scale bar: 100 μm. CA1, cornus ammoni 1; CA3, cornus ammoni 3; DG, dentate gyrus. (D) Immunofluorescence staining of ATRX (red) and DAPI (blue) in the medial prefrontal cortex (mPFC) of control and *Atrx*-cHet mice. Scale bar: 200 μm. (E) The percentage of ATRX-positive cells in the mPFC of control and *Atrx*-cHet mice (mean of three pairs±s.e.m., Student's two-tailed unpaired *t*-test). *P<0.05. (F) Haematoxylin and Eosin staining in the CA1, CA3 and mPFC of control and *Atrx*-cHet mice. Scale bar: 200 μm.

Conditional inactivation of *Atrx* is required to elucidate its functions in specific tissues, because general inactivation of the gene is embryonic lethal (Garrick et al., 2006). We thus generated mice with Cre recombinase-mediated deletion of *Atrx*-floxed alleles in the CNS using the *Nestin-Cre* driver line of mice. Hemizygous male mice (*Atrx*-cKO) died by postnatal day (P)1 (Fig. 1A). Owing to random X-inactivation in females, *Atrx* is expressed from only one of the alleles in any specific cell, resulting in a mosaic pattern of expression in the brain of *Atrx*-cHet mice (e.g. if the floxed allele is the active allele, these cells are functionally null for *Atrx*; however, if the floxed allele is the silent one, cells are functionally wild type for *Atrx*). This was validated by RT-qPCR with *Atrx* primers in exon 17 and the excised exon 18, showing ∼50% decreased *Atrx* expression in the cortex and hippocampus of *Atrx*-cHet mice compared with littermate controls (Fig. 1B). Moreover, a mosaic pattern of ATRX protein expression was observed by immunofluorescence staining of the hippocampus and medial prefrontal cortex (Fig. 1C,D). This was quantified in the medial prefrontal cortex in three pairs of control and cKO animals (Fig. 1E). Haematoxylin and Eosin staining of control and *Atrx*-cHet brain sections did not reveal major histological alterations in the CA1, CA3 and mPFC regions (Fig. 1F). These results demonstrated that inactivation of *Atrx* throughout the CNS was perinatal lethal but that *Atrx* deletion in approximately half of cells allowed survival of the female heterozygous mice to adulthood.

### Mosaic inactivation of *Atrx* in the CNS impedes normal body growth

**Fig. 2.**
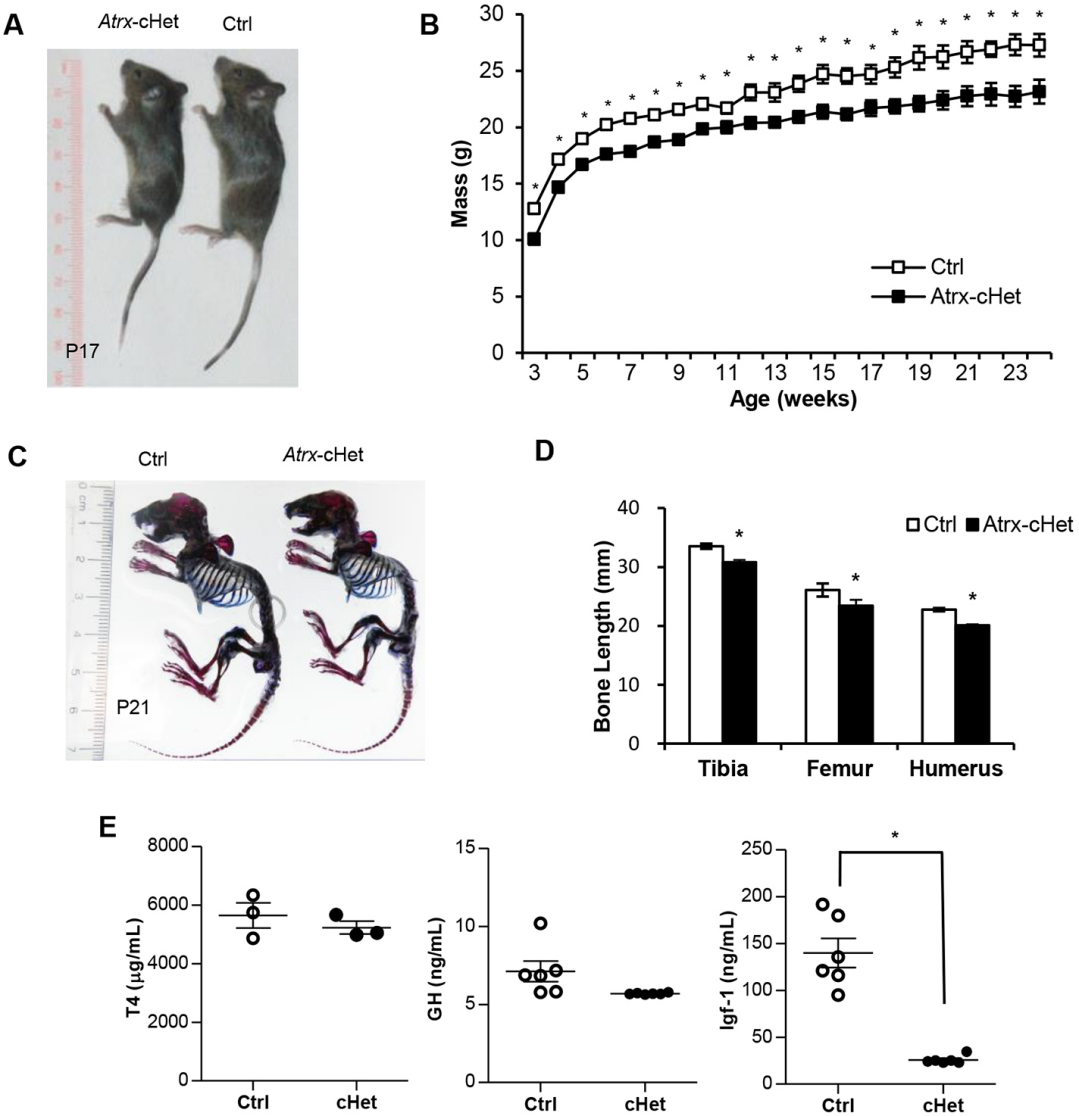
***Atrx*-cHet mice have reduced body weight and low circulating IGF-1.** (A) *Atrx*-cHet female mice are smaller than littermate-matched controls at P17. (B) Growth curve of mice from 3 to 24 weeks of age (*n*=13; *P<0.05). Data are represented as means±s.e.m., two-way repeated-measures ANOVA with Benjamini–Hochburg *post-hoc* test. (C) Skeletal stains of P21 control and *Atrx*-cHet mice showing cartilage (blue) and bone (red). (D) The lengths of long bones of *Atrx-*cHet mice (*n*=21) are decreased compared with control mice (*n*=19). (E) Circulating concentrations of T4, GH and IGF-1 in *Atrx*-cHet and control mice (*n*=3) at P17. Data are represented as means±s.e.m., Student's two-tailed, unpaired *t*-test. *P<0.05.

*Atrx*-cHet mice were weighed weekly for the first 24 postnatal weeks. The data showed that the *Atrx*-cHet mice weighed significantly less than control mice over this time period (*F*=17.87, *P*=0.0003; Fig. 2A,B). Alcian Blue and Alizarin Red skeletal staining of P17 mice reveal that the *Atrx*-cHet skeletons were smaller than those of the control mice (Fig. 2C). Tibia, femur and humerus bones were also measured and found to be significantly shorter in the *Atrx*-cHet mice compared with littermate controls (Fig. 2D).

We previously reported that deletion of *Atrx* in the developing mouse forebrain and anterior pituitary leads to low circulating concentrations of IGF-1 and thyroxine (T4) (Watson et al., 2013). Some evidence suggests that T4 regulates the prepubertal concentrations of insulin growth factor 1 (IGF-1), whereas after puberty this regulation is largely mediated by growth hormone (GH) (Xing et al., 2012). Given that the *Atrx*-cHet mice were smaller than control mice, we examined the concentrations of T4, IGF-1 and GH in the blood by enzyme-linked immunosorbent assasy (ELISAs). We observed no significant difference in T4 and GH concentrations between P17 *Atrx*-cHet mice and control littermates. However, there was a large (80%) and significant decrease in IGF-1 concentrations (Fig. 2E). Thus, the reduced body size of the *Atrx*-cHet mice was correlated with low circulating IGF-1 concentrations.

### Hindlimb-clasping phenotype in *Atrx*-cHet mice

**Fig. 3.**
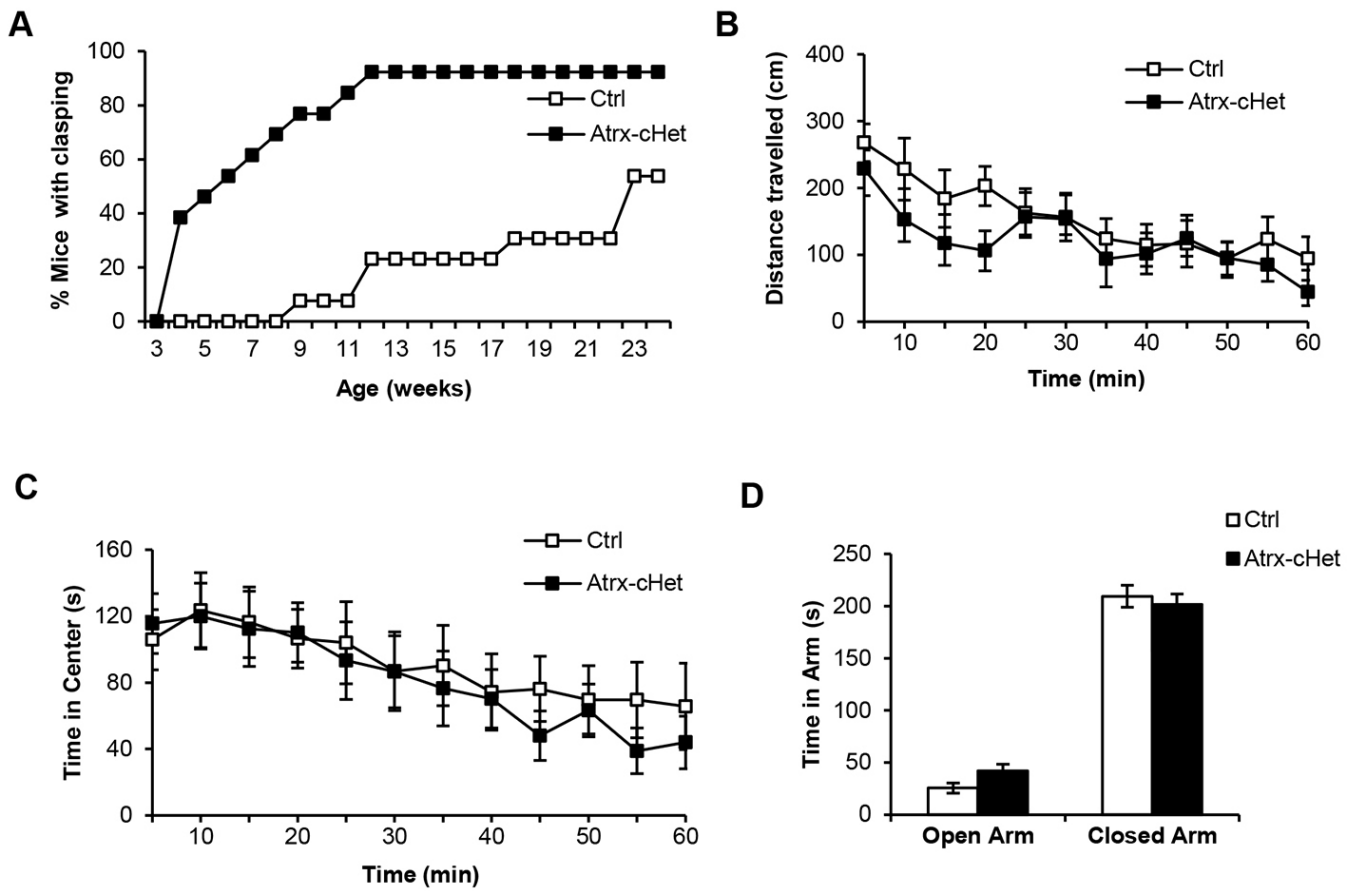
***Atrx*-cHet mice exhibit hindlimb clasping but normal activity and anxiety levels.** (A) Hindlimb clasping was evaluated in control (*n*=13) and *Atrx*-cHet (*n*=12) female mice and data plotted as the proportion of mice with hindlimb clasping from 3 to 25 weeks of age. (B,C) The open-field test showed no difference in distance travelled (B) and time spent in the centre (C) between control (*n*=11) and *Atrx*-cHet female mice (*n*=14). (D) Elevated plus maze test shows no difference in time spent in the open and closed arms in control (*n*=11) and *Atrx*-cHet (*n*=13) female mice. The data are represented as means±s.e.m. and a two-way ANOVA test was performed.

The *Atrx*-cHet mice displayed increased hindlimb clasping compared with control mice, with >90% exhibiting limb clasping by 3 months of age (*F*=20.78, *P*<0.0001; Fig. 3A). In the open-field test, the distance travelled was not significantly different between control and *Atrx*-cHet mice, indicating that activity and locomotion were normal (*F*=0.20, *P*=0.66; Fig. 3B). Anxiety levels were also normal, based on the time spent in the centre of the open-field apparatus (*F*=0.84, *P*=0.44; Fig. 3C). Likewise, their performance in the elevated plus maze revealed no significant difference in the amount of time that control and *Atrx*-cHet mice spent in the open versus closed arms (*F*=0.68, *P*=0.41; Fig. 3C,D). We concluded that the *Atrx*-cHet mice were not hyper- or hypo-active and did not exhibit excessive anxiety, but the increased level of hindlimb-clasping behaviour was suggestive of neurological defects.

### *Atrx*-cHet mice have normal working memory but deficits in object recognition memory

**Fig. 4.**
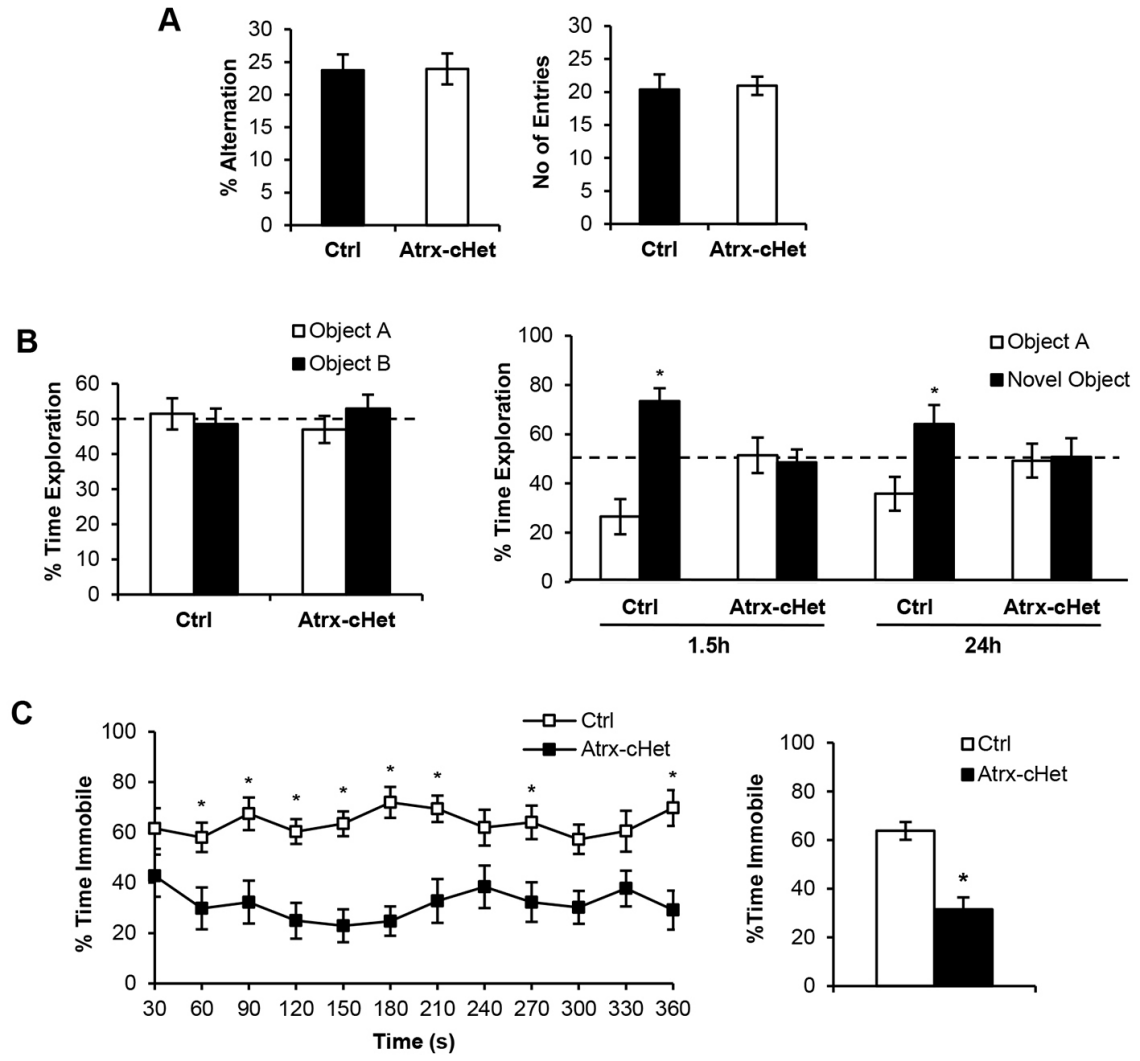
**Impaired novel object recognition and contextual fear memory in *Atrx*-cHet mice.** (A) Percentage alternation and number of arm entries in the Y-maze test by control (*n*=14) and *Atrx*-cHet (*n*=15) female mice. (B) Control (*n*=14) and *Atrx*-cHet (*n*=14) mice displayed similar preference for identical objects in the training session of the novel object recognition task. The *Atrx*-cHet mice failed to display a preference for the novel object 1.5 and 24 h later (*P<0.05). (C) *Atrx*-cHet (*n*=14) mice spent less time immobile than control mice (*n*=14) in the fear-conditioning paradigm. The total percentage of time spent immobile is shown on the right (*P<0.0001). Statistical analyses made use of Student's two-tailed, unpaired *t*-test or two-way ANOVA; data are represented as means±s.e.m. and *P<0.05.

Given that *ATRX* mutations are linked to ID, we next evaluated memory in *Atrx*-cHet mice using various established paradigms. We first tested short-term working memory in the Y-maze task (de Castro et al., 2009). No difference was detected between control and *Atrx*-cHet mice in the percentage alternation or in the number of entries into the arms, suggesting that working memory was normal in *Atrx*-cHet mice (*t*=0.05, *P*=0.96; Fig. 4A). We then tested the *Atrx*-cHet mice in the spontaneous novel object recognition task that mainly involves the prefrontal cortex and hippocampus (Ennaceur and Delacour, 1988). In rodents, the natural tendency to seek out and explore novelty leads to a preference for the novel over the familiar object, indicating recognition memory of the familiar object (Bevins and Besheer, 2006). During the habituation period, both control and *Atrx*-cHet mice spent ∼50% of the allotted time with each individual object (Fig. 4B). In the course of the short-term memory test (1.5 h), control mice spent ∼70% of their time with the novel object, whereas *Atrx*-cHet mice still spent ∼50% of their time with each object, suggesting an inability to remember the familiar object (Fig. 4B). Similar results were obtained in the long-term memory test (24 h). The total amount of time spent interacting with the objects was unchanged between control and *Atrx*-cHet mice during all three tests, ruling out visual or tactile impairment.

### *Atrx*-cHet mice display deficits in contextual fear and spatial memory

To evaluate contextual fear memory, mice were placed in a box with distinctive black and white patterns on the sides for 3 min and shocked after 2.5 min. Twenty-four hours later, they were placed back into the same box with the same contextual cues, and the time spent freezing was measured at 30 s intervals. The data showed that the *Atrx*-cHet mice spent less time freezing than control mice (*F*=28.57, *P*<0.0001), and the total percentage of immobility time was significantly lower for *Atrx*-cHet mice, indicating impaired fear memory in these mice (*t*=5.35, *P*<0.0001; Fig. 4C).

**Fig. 5.**
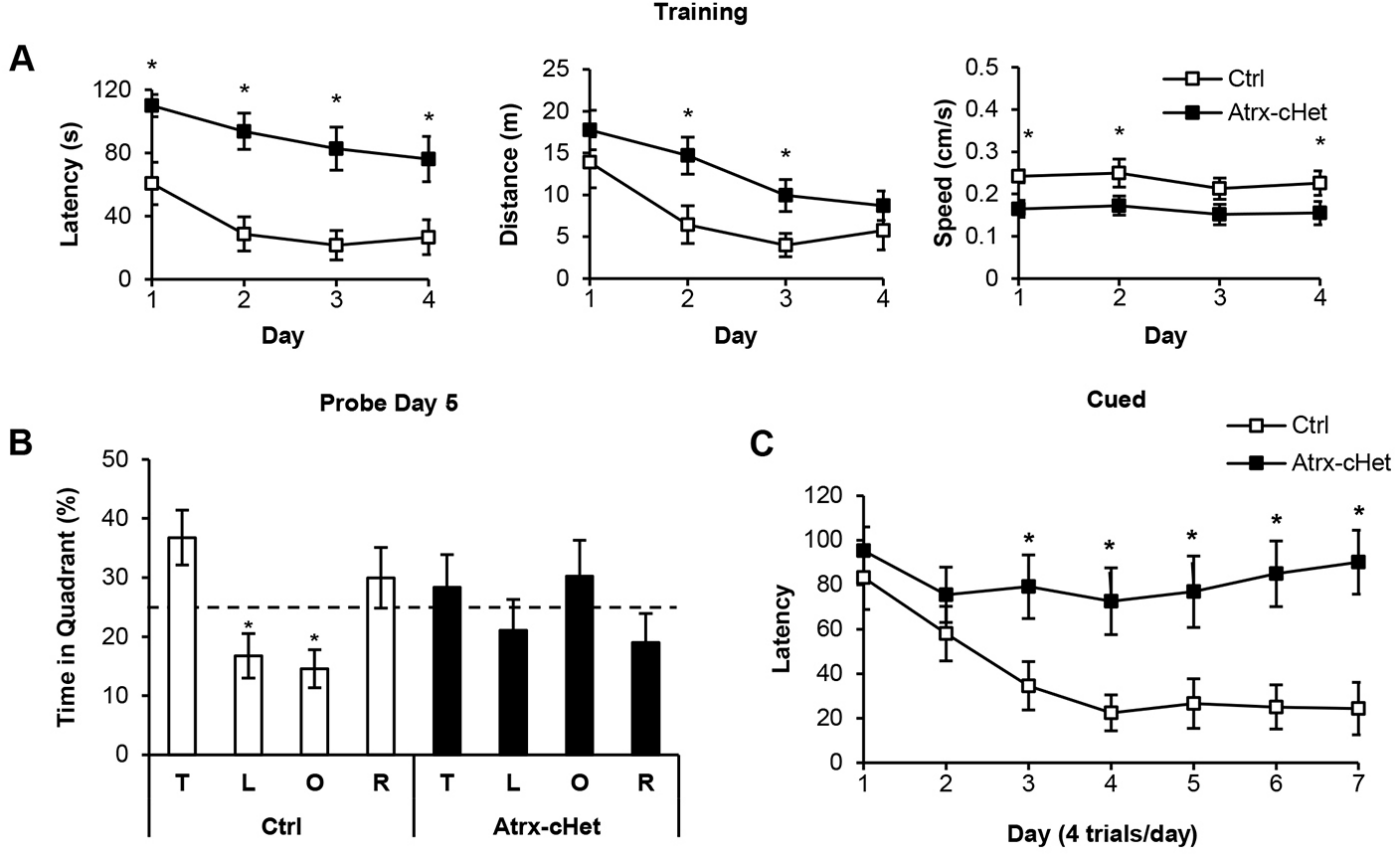
***Atrx*-cHet mice perform poorly in the Morris water-maze paradigm.** (A) *Atrx*-cHet mice (*n*=13) spent more time finding the platform compared with control mice (*n*=11) over the four consecutive days of training (*P<0.05). They swam longer distances but at a lower speed compared with control mice (*P<0.05). (B) Control mice spent more time swimming in the target quadrant (T) compared with the left (L) and opposite (O) quadrants (*P<0.05) on the day 5 probe test, whereas *Atrx*-cHet mice spent ∼25% of their time in each of the quadrants. (C) In the cued version of the Morris water maze, *Atrx*-cHet mice (*n*=11) were unable to learn the location of the visible platform, whereas the control mice could effectively learn this task (*n*=11). Data are represented as means±s.e.m. and a two-way ANOVA test was done. *P<0.05.

The Morris water maze was next used to evaluate hippocampus-dependent spatial memory (Morris, 1984). During the 4 days of training, the *Atrx*-cHet mice took significantly more time finding the target platform while swimming a longer distance compared with control mice (latency *F*=31.44, *P*<0.0001; distance *F*=12.29, *P*<0.01; Fig. 5A). The *Atrx*-cHet mice also swam more slowly than control mice (*F*=15.40, *P*<0.001; Fig. 5A). During testing on the fifth day, the platform was removed and the time spent in each quadrant recorded as a measure of spatial memory. Whereas control mice spent significantly more time in the target quadrant than the left or opposite quadrant (*F*=4.70, *P*<0.01), *Atrx*-cHet mice showed no preference for the target quadrant (*F*=0.75, *P*=0.53; Fig. 5B). The results suggested that spatial learning and memory might be impaired in the *Atrx*-cHet mice. The cued Morris water maze was used to determine whether motivational or sensorimotor defects contributed to the phenotype seen in the noncued version of the test. Whereas the control mice quickly learned to correlate the cue with the platform, the *Atrx*-cHet mice were unable to do so (*F*=14.09, *P*<0.01; Fig. 5C). We noticed that the mice failed to show normal signs of aversion to water during this task, with a preference to be swimming rather than to climb on the platform during training, even jumping back into the water after being placed on the platform.

### *Atrx*-cHet mice have normal motor endurance and motor memory

**Fig. 6.**
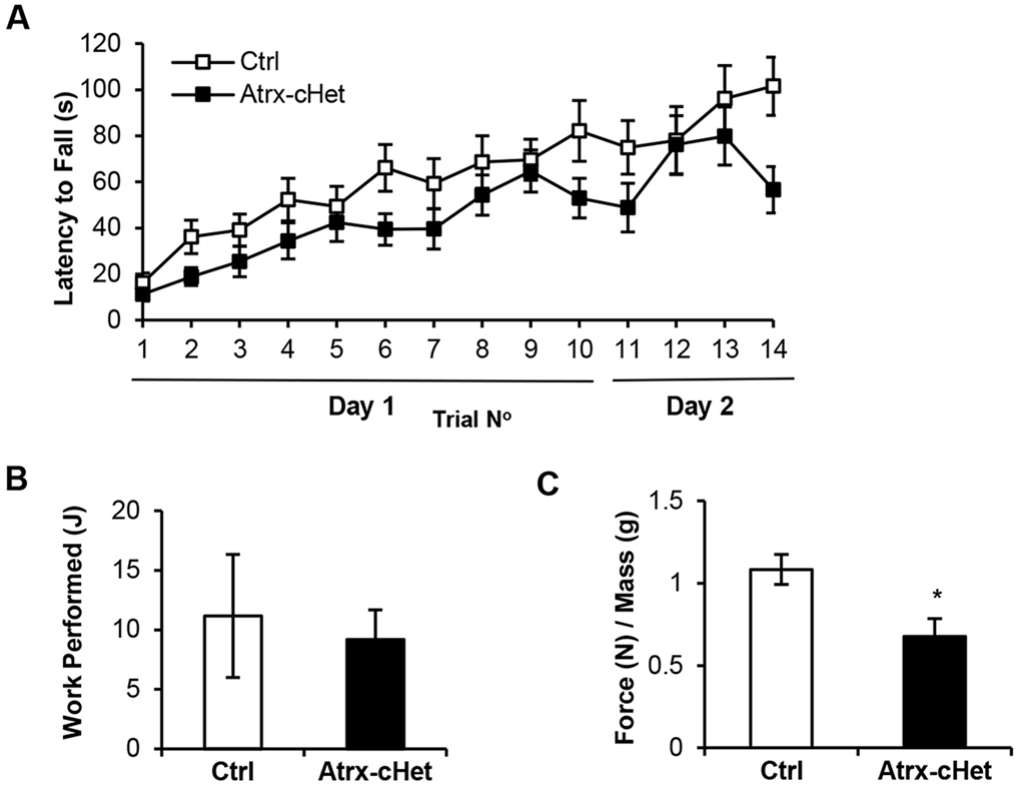
**Normal motor memory and endurance but decreased grip strength in *Atrx*-cHet mice.** (A) *Atrx*-cHet (*n*=18) and control (*n*=16) female mice performed normally in the Rotarod test. (B) *Atrx*-cHet (*n*=15) and control (*n*=15) mice exhibited similar performance in the treadmill test. (C) *Atrx*-cHet mice (*n*=13) exhibited a decreased grip strength compared with control mice (*n*=11), normalized to body mass. Statistical analyses made use of two-way ANOVA or Student's two-tailed, unpaired *t*-test; data are represented as means±s.e.m. *P<0.05.

Given that the *Atrx*-cHet mice swam more slowly than control animals in the Morris water-maze task, we considered that perhaps the test was confounded by deficits in motor skills. To clarify this issue, we examined endurance and motor skills further in the mutant mice. We found that motor function and balance measured in the Rotarod task were not significantly different in *Atrx*-cHet mice during any of the trials (*F*=3.02, *P*=0.09; Fig. 6A). *Atrx*-cHet mice also performed similarly to control animals in the treadmill task (*t*=0.34, *P*=0.73; Fig. 6B). By contrast, *Atrx*-cHet mice exhibited decreased forelimb grip strength, normalized to body weight (*t*=2.80, *P*<0.05; Fig. 6C).

## DISCUSSION

This study demonstrates that deletion of *Atrx* in the CNS leads to endocrine defects and behavioural abnormalities. Specifically, we see impairments in spatial learning and memory in the Morris water maze, in contextual fear conditioning and in novel object recognition.

We previously reported that mice lacking ATRX expression in the embryonic mouse forebrain have an average lifespan of 22 days (Watson et al., 2013). It is thus not surprising that inactivation of *Atrx* using the *Nestin*-*Cre* driver (which mediates deletion in the majority of CNS cells) is neonatal lethal. By contrast, the *Atrx*-cHet female mice survived to adulthood, probably because roughly half of all *Nestin*-expressing cells and their progeny are spared. Mosaic loss of ATRX in *Atrx*-cHet female mice still negatively affects development, as the mice are smaller compared with littermate controls, and the length of long bones is decreased. As the *Nestin*-*Cre* driver does not promote *Cre* expression in bone progenitors (Wiese et al., 2004), this phenotype might result from the low concentration of circulating IGF-1 in these mice. The reason for low IGF-1 is difficult to pinpoint in our mice. It has been shown that mice expressing *Cre* under the control of the *Nestin* promoter are smaller as a result of a decrease in mouse GH (Declercq et al., 2015). However, in our hands, GH concentrations are normal in the *Atrx*-cHet mice. Given the normal concentrations of both T4 and GH, there could be unanticipated expression of *Cre* in the liver that affects IGF-1 production. Examining the potential off-target expression of *Cre* will be required to elucidate the mechanism of IGF-1 downregulation in these mice.

The *Atrx*-cHet mice displayed a variety of behavioural abnormalities. We initially noticed that the mice displayed excessive hindlimb clasping, which could indicate neurological impairment (Guy et al., 2001). This prompted us to perform additional tests to assess neurobehaviour of the mice. We observed no change in general activity or anxiety using the open-field test and elevated plus maze, respectively, and no change in working memory in the Y-maze task. The *Atrx*-cHet mice exhibited increased latency to reach the platform in the Morris water-maze task, which might indicate a defect in spatial memory. However, the findings are difficult to interpret because *Atrx*-cHet mice swam at a lower speed, which could indicate a problem with their ability to swim rather than with memory. It was previously reported that mice lacking MeCP2 protein, an established interactor with ATRX in the brain, exhibit navigational difficulties in the Morris water maze (Stearns et al., 2007). Significant differences in swimming ability made it difficult to conclude whether the increased latency to the platform was attributable to motor or cognitive deficits, similar to our findings with the *Atrx*-cHet mice. Although we did not observe defective motor skills in the Rotarod or treadmill tests or decreased activity in the open-field test, we noticed that the mice failed to show normal signs of aversion to water during this task and preferred to be swimming rather than to climb on the platform during training, even jumping back into the water after being placed on the platform. We attempted to test the mice in the Barnes maze, another spatial learning and memory test, but the heterozygote mice tended to jump off the edge of the maze. Based on these observations, it will be important in the future to perform additional tests that gauge the level of motivation in these mice.

Despite these issues, which might require further experimentation for a full understanding, we obtained supporting evidence that memory is affected in the *Atrx*-cHet mice in the contextual fear and the novel object recognition tasks. Additional support comes from a previous study done in a mouse model of Chudley–Lowry syndrome associated with reduced expression of ATRX (Nogami et al., 2011). The authors of that study reported an impairment in contextual fear memory and suggested that ATRX might play a role in regulation of adult-born neurons. Our results show defects not only in contextual fear memory, but also in novel object recognition and, potentially, the Morris water-maze task. This might indicate a role for ATRX not only in adult-born neurons, but perhaps also in the amygdala, hippocampus and the rest of the medial temporal lobe, structures which are vital for the tasks impaired in the *Atrx*-cHet mice (Phillips and LeDoux, 1992; Logue et al., 1997; Wan et al., 1999).

The DAXX protein is a well-established interactor with ATRX. Although the behaviour of DAXX knockout mice has not yet been investigated, a study previously demonstrated that DAXX binds with ATRX to the promoters of several immediate-early genes upon activation of cortical neuronal cultures (Michod et al., 2012). DAXX was also shown to be crucial for the incorporation of histone H3.3 at these gene promoters, supporting a potential role for DAXX and ATRX the initial steps of memory consolidation. EZH2, a member of the PRC2 complex that mediates H3K27 trimethylation, is another putative binding partner of ATRX (Cardoso et al., 1998; Margueron et al., 2009). Inducible deletion of the *Ezh2* gene in neural progenitor cells in the adult brain caused impaired spatial learning and memory and contextual fear memory, suggesting that EZH2 (potentially with ATRX) provides important cues in adult neural progenitor cells (Zhang et al., 2014).

We emphasize that these mice do not model the ATR-X syndrome, where only males are affected and females exhibit 100% skewing of X-chromosome inactivation and are therefore largely unaffected. Rather, the *Atrx*-cHet mice are a useful tool to probe ATRX function in the CNS. Overall, our study presents compelling evidence that ATRX is required in the mouse CNS for normal cognitive processes and sets the stage for additional investigations delving into the mechanisms by which it regulates chromatin structure and gene expression in neurons in the context of learning and memory.

## MATERIALS AND METHODS

### Animal care and husbandry

Mice were exposed to a 12 h:12 h light–dark cycle and with water and chow *ad libitum*. The *Atrx^loxP^* mice have been described previously (Bérubé et al., 2005). *Atrx^loxP^* mice (129svj) were mated with mice expressing Cre recombinase under the control of the *Nestin* gene promoter (Bl6) (Tronche et al., 1999). The progeny include hemizygous male mice that produce no full-length ATRX protein in the CNS (*Atrx^f/y^ Cre^+^*) and heterozygous female mice with approximately half the cells lacking ATRX protein as a result of the random pattern of X-inactivation (*Atrx^f/+^ Cre^+^*). Male and female littermate floxed mice lacking the *Cre* allele were used as controls. Genotyping of tail biopsies for the presence of the floxed and *Cre* alleles was performed as described previously (Bérubé et al., 2005; Seah et al., 2008). All procedures involving animals were conducted in accordance with the regulations of the Animals for Research Act of the province of Ontario and approved by the University of Western Ontario Animal Care and Use Committee (2008-041). Behavioural assessments started with less-demanding tasks (grip force, open-field tests), followed by more-demanding ones (Morris water maze). Experimenters followed ARRIVE guidelines: mouse groups were randomized, they were blind to the genotypes, and software-based analysis was used to score mouse performance in most of the tasks. All experiments were performed between 09.00 and 16.00 h.

### Immunofluorescence staining

Mice were perfused and the brains fixed for 72 h with 4% paraformaldehyde in PBS and cryopreserved in 30% sucrose/PBS. Brains were flash frozen in Cryomatrix (Thermo Fisher Scientific) and sectioned as described previously (Ritchie et al., 2014). For immunostaining, antigen retrieval was performed by incubating slides in 10 mM sodium citrate at 95°C for 10 min. Cooled slides were washed and incubated overnight in anti-ATRX rabbit polyclonal antibody (Santa Cruz Biotechnology, SC-15408; 1:200, H-300) (Watson et al., 2013) diluted in 0.3% Triton-X100 in PBS. Sections were washed and incubated with goat anti-rabbit Alexa Fluor 594 (Life Technologies) for 1 h. Sections were counterstained with DAPI and mounted with SlowFade Gold (Invitrogen). Cell counts were done in three control–KO littermate-matched pairs in a blinded manner.

### Microscopy

All images were captured using an inverted microscope (DMI 6000b; Leica) with a digital camera (ORCA-ER; Hamamatsu). Openlab image software was used for manual image capture, and images were processed using the Volocity software (PerkinElmer).

### Haematoxylin and Eosin staining

Brain cryosections (8 μm thick) from 3-month-old mice were rehydrated in 70% ethanol for 2 min followed by tap water for 5 min. They were then placed in CAT Haematoxylin (Biocare) for 2 min, placed under running tap water for 1 min, and set in filtered Tasha's Bluing Solution (Biocare) for 30 s. The slides were placed under running tap water for 10 min and set in filtered Eosin Y (Fisher Scientific) for 2 min. Immediately afterwards, the cells were dehydrated in 70% ethanol for 30 s each, then 90% ethanol for 1 min and 100% ethanol for 2 min each. The slides were placed in xylene 3× for 5 min and mounted with Permount (Fisher Scientific) immediately after.

### RT-qPCR

Total RNA was isolated from control and *Atrx*-cHet rostral cortex and hippocampus using the RNeasy Mini Kit (Qiagen) and reverse transcribed to cDNA using 1 μg RNA and SuperScript II Reverse Transcriptase (Invitrogen). cDNA was amplified in duplicate using primers in the following conditions: 95°C for 10 s, 55°C for 20 s and 72°C for 30 s for 35 cycles. Primers detected *Atrx* exons 17 and 18. Standard curves were generated for each primer pair. Primer efficiency (*E*) was calculated as *E*=(10^−1/slope^−1)×100%, where a desirable slope is −3.32 and *R*^2^>0.990. All data were corrected against β-actin.

### ELISAs

Blood was collected from the inferior vena cava of P17 mice. One hundred microlitres of 0.5 M EDTA pH 7.0 per millilitre of blood collected was added to the blood sample and centrifuged at 21,000 ***g*** for 10 min at 4°C. Plasma supernatant was collected and kept frozen at −80°C. Plasma IGF-1 concentration was measured using a mouse IGF-1 ELISA kit (R&D Systems, MG100). Plasma GH (Millipore, EZRMGH-45K) and T4 (Calbiotech, T4044T) were also measured by ELISA according to the manufacturers' instructions.

### Bone staining and measurements

Skinned and eviscerated P17 mouse carcasses were fixed overnight in 95% ethanol and transferred to acetone overnight (Ulici et al., 2009). Fixed skeletons were stained in a 0.05% Alizarin Red, 0.015% Alcian Blue, 5% acetic acid in 70% ethanol solution for 7-14 days. Stained skeletons were cleaned in decreasing concentrations of potassium hydroxide (2, 1 and 0.5%) for several days and stored in 50:50 70% ethanol/glycerol solution. Alcian Blue and Alizarin Red-stained skeletons were imaged using an Olympus SP-57OUZ digital camera. The lengths of the tibia, femur and humerus, the width of the skull and the length of the foot from at least four different littermate pairs from both mouse models were imaged using the Zeiss Stereo Zoom Microscope Stemi SV6 and measured with a ruler accurate to 0.1 mm.

### Hindlimb clasping, grip force, Rotarod, treadmill and open-field tests

Hindlimb clasping was measured by lifting mice up by the base of the tail. Clasping was scored on a scale of 0 (no clasping, limbs splayed) to 2 (clasping, wringing paws).

Grip force, an indicator of muscular strength, was measured using a Grip Strength Meter (Columbus Instruments) (Solomon et al., 2013). The meter was positioned horizontally, and the mice were held by the base of the tail and lowered towards the triangular pull bar. Once the mice had gripped the bar, the meter was calibrated, and the mice were gently pulled away from the apparatus. The force applied to the bar as the mice released it was recorded as peak tension (in newtons). This test was repeated five times, with the highest and lowest value being removed for user error, and the remaining three values were averaged for the final grip strength.

For the Rotarod task, mice were placed on the Rotarod apparatus (San Diego Instruments) and rotation was increased from 5 to 35 rpm over 5 min. Latency to fall was recorded automatically. Ten trials were performed on the first day and four were performed on the second day. There was an inter-trial period of 10 min, during which the mice were placed in their home cage.

Training for the treadmill test occurred over 4 days (3 min day^−1^). On the first day, the incline was set to 5° and increased by 5° every day to a maximum of 20°. The initial speed was 8 m min^−1^, and the treadmill was accelerated by 1 m min^−2^. On the subsequent training days 2, 3 and 4, the initial speed was increased to 10, 11 and 12 m min^−1^, respectively, with constant acceleration. During testing on the fifth day, the initial speed was 12 m min^−1^ and accelerated to 20 m min^−1^ over the course of 15 min. Distance to exhaustion was measured, and the work performed (*W*, in joules) was calculated using the formula: *W* (J)=body weight (kg)×cos20°×9.8 (J kg^−1^×m)×distance (m).

In the open-field test, locomotor activity was automatically recorded (AccuScan Instrument). The mice were placed in an arena with an area of 20 cm×20 cm with 30-cm-high walls. Mice were acclimated to the locomotor room for 10 min before testing. Locomotor activity was measured in 5 min intervals over 2 h, as previously described (Martyn et al., 2012).

### Elevated plus maze, Y-maze, fear conditioning and novel object recognition

Animals were placed in the centre of the elevated plus maze (Med Associate) and their activity was recorded over 5 min. The total time spent in the open and closed arms was recorded using computer software (AnyMaze). The centre of the mouse body was used as an indicator of which zone they were in.

Spontaneous Y-maze alternation was measured using a symmetrical three-armed Y-maze as described (de Castro et al., 2009). Video tracking was performed using computer software (AnyMaze) and the order and number of entries into each arm recorded. Each mouse underwent one trial lasting 5 min. Spontaneous alternation was counted when a mouse entered all three arms in a row without visiting a previous arm.

To measure contextual fear, mice were placed in a 20 cm×10 cm clear acrylic enclosure with a metal grid floor and one wall distinct from the others (stripes were drawn on one of the walls). The chamber was equipped with an electric shock generator. Videos were recorded using the AnyMaze video tracking software. On Day 1, mice were allowed to explore the enclosure freely, and at 150 s the mice were given a shock (2 mA, 180 V, 2 s). Shock sensitivity was confirmed by vocalization of the mice. Thirty seconds later the mice were returned to their home cage. After 24 h, the mice were placed back into the enclosure for 6 min and freezing time was measured in 30 s intervals. Freezing was defined as immobility lasting >0.5 s.

To test novel object recognition, mice were habituated with no objects in an open arena (40 cm×40 cm) for 5 min on both Day 1 and Day 2. On Day 3, mice were placed in the arena with two identical objects (A; a red plastic ball attached on top of a yellow cube base) and allowed to explore for 10 min. Video tracking was used (AnyMaze). To test short-term memory, 1.5 h after training the mice were placed back in the arena for 5 min with one previous object (A) and one novel object (B; a blue plastic pyramid attached on top of a green prism base). To test long-term memory, 24 h after training the mice were placed back in the arena for 5 min with one previous object (A) and one novel object (B). Recognition of previous and novel objects was expressed as the percentage of time spent with each object compared with the total time interacting. Interaction with the object was defined as sniffing or touching the object, but not leaning against or standing on the object.

### Morris water maze

The Morris water-maze test was conducted as described previously (Vorhees and Williams, 2006). Mice were given four trials (90 s) a day for 4 days consecutively, with a 15 min inter-trial period. The latency to find the platform was recorded using video-tracking software (AnyMaze). If the mice did not find the platform during the 90 s, they were gently guided onto the platform. On the 5th and the 12th day, the platform was removed and time spent in each quadrant of the maze recorded using the video software. The task was performed in a pool 1.5 m in diameter with 25°C water, and the platform was submerged 1 cm beneath the water surface. Spatial cues (shapes printed in black and white) were distributed around the pool. For the cued version of the Morris water maze, mice were subjected to four trials (90 s) per day for 7 days consecutively, with a 30 s inter-trial period. If the mouse did not find the platform after 90 s, it was gently guided onto the platform. The visible platform and the mouse starting location changed with each trial, so they were unique between trials. The platform was made visible by placing a red plastic cube on top of the platform, which was wiped with ethanol between each trial.

### Statistical analyses

All data were analysed using GraphPad Prism software or SPSS, with Student's *t*-test (unpaired, two tailed) or one- or two-way ANOVA with Bonferroni or Benjamini–Hochburg correction where indicated. All results are depicted as means±s.e.m. unless indicated otherwise. Values of *P*≤0.05 were considered to indicate significance.
